# Root Causes of Fungal Coinfections in COVID-19 Infected Patients

**DOI:** 10.3390/idr13040093

**Published:** 2021-12-04

**Authors:** Arman Amin, Artin Vartanian, Nicole Poladian, Alexander Voloshko, Aram Yegiazaryan, Abdul Latif Al-Kassir, Vishwanath Venketaraman

**Affiliations:** 1 College of Osteopathic Medicine of the Pacific, Western University of Health Sciences, Pomona, CA 91766-1854, USA; arman.amin@westernu.edu (A.A.); nicole.poladian@westernu.edu (N.P.); alexander.voloshko@westernu.edu (A.V.); aram.yegiazaryan@westernu.edu (A.Y.); abdullatif.alkassir@westernu.edu (A.L.A.-K.); 2 School of Medicine, St. George’s University, St. George’s 999166, Grenada; avartani@sgu.edu

**Keywords:** COVID-19, fungal infection, Aspergillosis, Candidiasis, Cryptococcosis, co-infection risk factors

## Abstract

COVID-19 is caused by severe acute respiratory syndrome coronavirus 2 (SARS-CoV-2) and has infected over 200 million people, causing over 4 million deaths. COVID-19 infection has been shown to lead to hypoxia, immunosuppression, host iron depletion, hyperglycemia secondary to diabetes mellitus, as well as prolonged hospitalizations. These clinical manifestations provide favorable conditions for opportunistic fungal pathogens to infect hosts with COVID-19. Interventions such as treatment with corticosteroids and mechanical ventilation may further predispose COVID-19 patients to acquiring fungal coinfections. Our literature review found that fungal coinfections in COVID-19 infected patients were most commonly caused by *Aspergillus*, *Candida* species, *Cryptococcus neoformans*, and fungi of the *Mucorales* order. The distribution of these infections, particularly *Mucormycosis*, was found to be markedly skewed towards low- and middle-income countries. The purpose of this review is to identify possible explanations for the increase in fungal coinfections seen in COVID-19 infected patients so that physicians and healthcare providers can be conscious of factors that may predispose these patients to fungal coinfections in order to provide more favorable patient outcomes. After identifying risk factors for coinfections, measures should be taken to minimize the dosage and duration of drugs such as corticosteroids, immunosuppressants, and antibiotics.

## 1. Introduction

The winter of 2019 marked the initial spread of the COVID-19 outbreak, caused by severe acute respiratory syndrome coronavirus 2 (SARS-CoV-2) [1]. Coronavirus is categorized as an RNA virus within the subfamily coronaviridae [2]. The novel zoonotic outbreak has been traced to Wuhan, China, starting in December of 2019. According to the Centers for Disease Control and Prevention (CDC), the COVID-19 pandemic has resulted in over 35 million cases, and over 611 thousand deaths in the United States (US) alone, as of August 2021 [3]. Individual and environmental factors play a role in an individual’s susceptibility to COVID-19 [4]. The CDC has reported an increased risk of sickness and death among racial and ethnic minorities, disabled individuals, and older adults, as 95% of deaths are among individuals over the age of 45 [5].

The spread of COVID-19 occurs both via cross-species and human-to-human interaction [2]. Transmission of COVID-19 occurs via respiratory droplets and aerosols containing the virus, along with direct contact transmission [6,7]. These forms of transmission include but are not limited to close contact with individuals sneezing or coughing, contact with host mucosal membranes of eye, nose, mouth, and medical procedures such as bronchoscopy that generate aerosols [8]. With an incubation period of 5–14 days, COVID-19 is seen to be spread by both infected asymptomatic and symptomatic individuals [9]. Symptoms of COVID-19 include fever, cough, dyspnea, fatigue, shortness of breath, muscle aches, among other manifestations [10,11].

Viral binding to the host target cell results in interleukin-6 (IL-6) production and the activation of the nuclear factor kappa B (NF-κB) pathway, resulting in a proinflammatory state characterized by an increase in macrophage and cytokine concentrations. The presenting cytokine storm and immune dysregulation of COVID-19 may develop acute respiratory distress syndrome, organ failure, coagulation, and more [9]. This cytokine storm response can lead to T-cell exhaustion, seen often in chronic infectious states. Patients with COVID-19 have been found to have decreased levels of CD4+ T-lymphocytes (< 200 cells/μL), which increases susceptibility for fungal infection development [12,13]. Given that CD4+ T-lymphocytes play a role in the effective immune response to presenting pathogens, they indicate a patient’s immunologic status and functioning [14,15].

Research suggests that viral respiratory diseases, such as COVID-19 may predispose an individual to other fungal, bacterial, and viral coinfections and superinfections [16,17]. Superinfection, occurring subsequently, and coinfection, occurring concomitantly, cause greater difficulty and complication in diagnosis due to an overlap of symptoms and consequently complicate the treatment of COVID-19 [Table 1]. Such multi-infectious states often rtesult in a worse outcome than either infection alone [18,19]. Fungal infections, for instance, often have similar symptoms to COVID-19, such as cough, shortness of breath, and fever, making it difficult to distinguish between the two diseased states [20,21]. A summary of such symptoms has been provided in Table 1. Common fungal infections seen associated with COVID-19 infection include Aspergillosis, Candidiasis, Cryptococcosis, and Mucormycosis [21]. These infections are caused by fungi *Aspergillus genera*, *Candida Auris, Cryptococcus neoformans,* and fungi of Mucorales order, respectively. Fungi cause a variety of diseases in both immunocompetent and immunocompromised individuals. Fungal infections can develop as primary or secondary to other diseases, with modes of infection and risk varying with the pathogenic fungi that ultimately result in activation of the immune system [22]. A multi-infected state may function to increase systemic inflammation and consequently prolong recovery, leading to increased use of treatment methods, need for intensive care, and risk of death [23].

This review seeks to analyze literature regarding occurrences and mechanisms of fungal coinfection with COVID-19. We will characterize four common fungal infections and explore specific factors which predispose COVID patients to these infections. Understanding the contributing factors to increased multi-infectious states may guide clinical measures to reduce the risk of coinfection.

## 2. Methods

**Figure 1:**
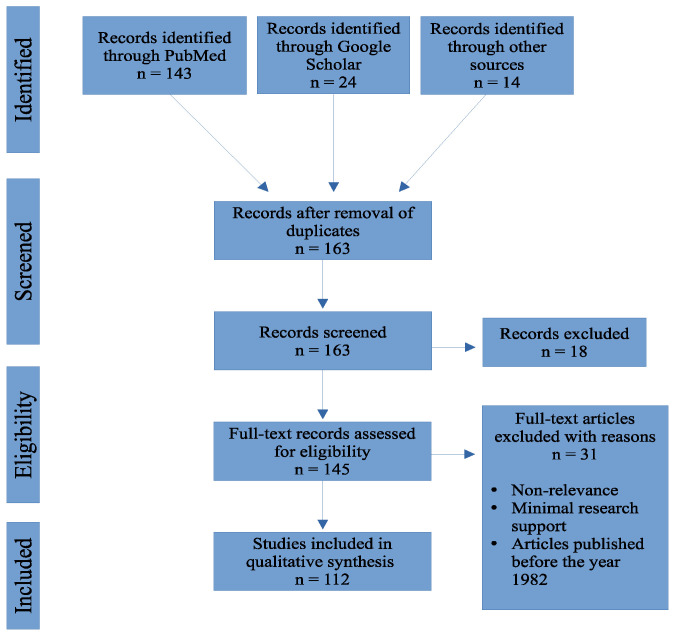
Flow chart outlining the number of articles accessed from online biomedical databases.

We searched online biomedical databases such as PubMed, The Lancet, and Google Scholar for journal articles. The first search included “COVID-19” and “fungal infections”. From the search, possible explanations for the increased incidence of fungal coinfections were explored. We focused our search on risk factors that had sufficient evidence and excluded risk factors with minimal evidence. Search criteria regarding publication timing were limited to the onset of the global pandemic (2019) to current studies. Supplemental data and information published prior to 2019 were considered as needed to expound upon fungal coinfection mechanisms. Figure 1 illustrates the flow diagram for study selection.

## 3. Countries with Cases of Fungal Infections

**Figure 2:**
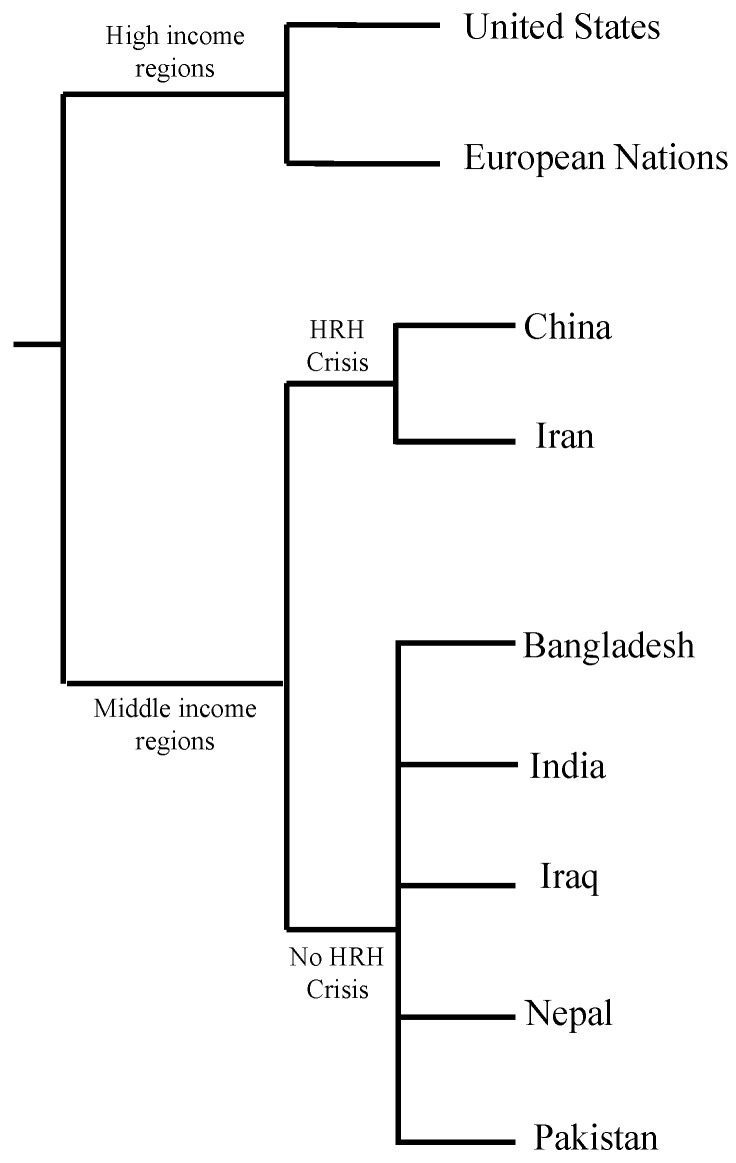
Income grouping of countries facing human resources for health crisis (HRH) according to World Health Organization (WHO) [32,33,34,35].

Opportunistic fungal coinfections in patients infected with COVID-19 have been documented across continents. At first, China reported fungal coinfections in patients who were ventilated for COVID-19. Europe and America also reported COVID-19 associated mucormycosis (CAM). From April 2020 to September 2020, Iran reported 15 CAM cases, most of them rhino-orbital mucormycosis. Reports of CAM cases have been documented in Iran, Pakistan, Nepal, Bangladesh, Iraq, as well as other countries [29]. In recent literature, India reported significantly higher cases of mucormycosis/black fungus coinfections in critically ill patients and patients recovering from COVID-19. The prevalence of mucormycosis is roughly 80 times higher in India than in other developing countries [30]. One of the reasons for the significantly higher prevalence of CAM is the burden of diabetes mellitus in low- and middle-income countries. Examples of low- and middle-income countries are referenced in Figure 2. A 2019 paper showed that four out of five people with diabetes live in low- and middle-income countries [31]. Complications of uncontrolled diabetes mellitus can explain the increased prevalence of fungal coinfections in low- and middle-income countries, as hyperglycemia acts as an immunosuppressant.

## 4. Root Cause of Coinfection

**Figure 3:**
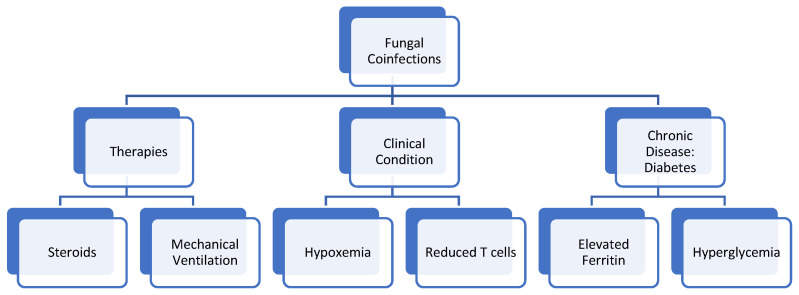
Therapies, clinical conditions, and pre-existing conditions that put patients with COVID-19 at risk for developing Fungal Coinfections.

Researchers and physicians alike believe that fungi can infect and germinate individuals with COVID-19 due to the ideal environment they present, which include: low oxygen states secondary to the patients’ hypoxemia, high glucose presence secondary to diabetes (well-known risk factor) and/or steroid-induced hyperglycemia, decreased phagocytic action of white blood cells secondary to the immunosuppression from the virus and/or steroid treatment, acidic environment from possible diabetic ketoacidosis (DKA) or metabolic acidosis, and high iron levels secondary to increased ferritin levels [36]. Coupled with the ideal environment caused by the COVID-19 virus, a prolonged hospitalization period and possible need for mechanical ventilation act as risk factors that facilitate the germination of fungi in COVID-19 patients. A retrospective cohort study done in Wuhan, China, analyzing 52 critically ill patients, identified that patients who had long hospital stays (>2 weeks), especially those who were admitted to the intensive care unit (ICU) and required mechanical ventilation, had a greater likelihood to develop a fungal coinfection. The study found that 3/52 patients, or 5.8%, had a fungal coinfection, including *A. flavus*, *A. fumigatus*, and *C. albicans* [37]. Many similar studies, which will be further discussed throughout the paper, describe the widespread prevalence and high mortality of fungal coinfections. This paper will now further dive into the specific ways in which COVID-19 infection may allow for fungal coinfection to occur. Figure 3 serves as a guide to the root causes of the coinfections explored in this paper.

### 4.1. Oxygen/Hypoxia Induced

Patients infected with COVID-19 commonly present with hypoxemia [38,39]. Possible clinical manifestations of hypoxemia include, but are not limited to, cyanosis, dyspnea, tachypnea, and tachycardia, secondary to lack of oxygen content in blood or lack of tissue oxygenation [40]. In contrast, some patients with COVID-19 induced hypoxia may show minimal to no symptoms of being in a hypoxic state, referred to as silent hypoxia. One study analyzes the possibility that inflammation and capillary damage resulting from COVID-19 infection interfere with blood and tissue oxygenation, resulting in the clinical development of hypoxia [38]. Fungal pathogens involved in the development of Aspergillosis, Candidiasis, Cryptococcosis, Mucormycosis, and various other fungal pathogens, have developed varying adaptations to allow their survival in hypoxic host environments, further enabling the possibility of COVID-19 and fungal coinfection. Oxygen necessity and consumption by both host and pathogen further contribute to developing a hypoxic environment [41]. Although COVID-19 is seen to result in the secondary development of hypoxia, research indicates the possibility that hypoxia-inducible factor-1α (HIF-1α), involved in mammalian response to hypoxia may be protective against COVID-19 pathogenesis due to its involvement in the downregulation of ACE-2 expression [42,43,44]. ACE-2 receptor has been identified as the point of entry for COVID-19. Therefore, any disruption in high-affinity binding between COVID-19 and ACE-2 receptors may reduce pathogenicity [45,46].

### 4.2. Diabetes

Diabetes Mellitus (DM) is one of the most well-established comorbidities of COVID-19, present in nearly 10% of hospitalized patients in international studies and one-third of patients hospitalized in New York City [47]. It has been well-linked to increased disease severity among COVID-19 patients, with uncontrolled hyperglycemia increasing the rate of hospitalization among individuals affected with COVID-19, as well as the mortality rate of those hospitalized [47,48]. Among diabetics admitted for COVID-19, those with uncontrolled blood glucose levels throughout the hospitalization saw significantly higher increases in mortality than those whose hyperglycemia was effectively controlled [49].

Several factors can explain the increased mortality among COVID-19 patients with uncontrolled diabetes. Firstly, hyperglycemia is a well-known immunosuppressant, inhibiting the host immune response via several mechanisms, including glycosylation of complement proteins leading to impaired function, impaired binding of oligosaccharides by C-type leptin (a process necessary for several immune functions), impaired opsonophagocytosis, and decreased production of Tumor necrosis factor alpha (TNF-a), Interleukin 10 (IL-10), and Interferon gamma (IFN-ℽ) [47,50,51,52]. It has also been proposed that hyperglycemia impairs T helper 1 (Th1) cell-mediated immunity, leading to accentuated inflammatory response and decreased antiviral response in COVID-19 patients [47].

Hyperglycemia has also been shown to nearly double the risk of superinfection and coinfection in COVID-19 patients [53]. While diabetes increases the risk for various infections, Mucormycosis and Candidiasis show a particularly strong association with the disease. In one review analyzing 101 COVID-19 associated Mucuormycocis infections, 80% of infected patients had pre-existing DM [36]. This relationship is believed to be due to hyperglycemia-induced DKA leading to increased levels of free iron in the host, which is particularly advantageous to the Mucormycosis-causing fungi [54]. In the case of Candida infections, hyperglycemia leads to the activation of a glucose-inducible protein which facilitates fungal adhesion to host tissue and subverts phagocytosis [55].

**Figure 4:**
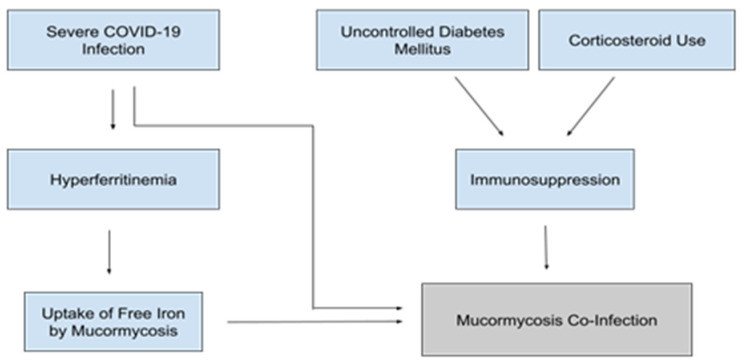
Factors contributing to the pathogenesis of Mucormycosis coinfections in patients with severe COVID-19 [54,63,64,65,66,67,68].

Given the increased mortality rate and risk for hospital-acquired superinfection, screening for undiagnosed diabetes is vital in caring for patients hospitalized with COVID-19. Furthermore, tight inpatient glucose control should be maintained as much as possible to limit hyperglycemia-mediated immunosuppression and secondary infection (Figure 4).

### 4.3. Steroids

Corticosteroids are used to treat various inflammatory conditions and autoimmune diseases. During the SARS outbreak from 2002–2004, steroids were used to minimize the deterioration of patients’ clinical condition by reducing the immune response [56]. A 2020 study found that ciclesonide, an inhaled corticosteroid, was specifically effective in suppressing the viral load of COVID-19, as they shorten intensive care unit (ICU) stay, stabilize hemodynamics, and shorten ventilation use in patients with COVID-19 [57]. A study conducted across ICUs in Brazil showed that intravenous dexamethasone in combination with standard care resulted in a statistically significant number of days free of ventilation in COVID-19 infected patients [58].

However, the use of steroids in hospitalized COVID-19 patients poses significant risk due to both their immunosuppressive effects and the associated risk of hyperglycemia. Both of these properties lead to an increased risk for secondary infections [53]. A 2020 review found a 3.33-fold increase in the development of invasive fungal infections in patients who received corticosteroid therapy compared to patients who did not receive steroids [59]. Prolonged use of steroids, in particular, may be associated with an increased risk of infections [56].

The benefits of steroid therapy in COVID-19 patients have been debated. Researchers evaluated the use of corticosteroid therapy in 409 patients with COVID-19 and found that the 28-day mortality increased and viral clearance decreased [60]. In contrast, the RECOVERY trial, which sampled 6245 patients, showed dexamethasone to decrease mortality in patients hospitalized with COVID-19 by 17% and by 36% in the subsets of patients who required invasive mechanical ventilation [61]. It has been proposed that the negative effects of steroid therapy are due to the lack of management of steroid-induced hyperglycemia, which negates the positive immunomodulatory effects of corticosteroid therapy [62]. Clinicians should be mindful of the potential adverse effects when evaluating their patients’ candidacy for steroid therapy. If clinicians do opt for steroid therapy, tight blood glucose control should be emphasized to minimize steroid-induced hyperglycemia. They must also be mindful of the increased risk for secondary infections and remain vigilant for any signs of secondary infection in patients who receive steroid therapy (Figure 4).

### 4.4. Ferritin and Free Iron Levels

Ferritin is a well-known acute-phase reactant, with increased Ferritin levels being shown to strongly correlate with disease severity in COVID-19 patients. Ferritin is a key mediator of immune dysregulation in these patients and has direct immune-suppressive and proinflammatory effects that contribute to the COVID-19 cytokine storm [63]. Ferritin levels are also known to be elevated in diabetic patients, which may partially explain the increased propensity for severe inflammatory reactions and death seen in hyperglycemic COVID-19 patients [64]. It has also been hypothesized that iron dysregulation seen in COVID patients leads to an excess of free iron, which leads to further toxicity from reactive oxygen species generation, increased inflammation, and thrombosis [65].

As mentioned in the section on diabetes, it is believed that hyperglycemia and DKA lead to increased Mucormycosis susceptibility in large part through the increase of unbound serum iron [54]. Thus, high iron states secondary to severe COVID infection may increase Mucormycosis susceptibility even in the absence of hyperglycemia or DKA (Figure 4). Increased levels of free iron could lead to susceptibility to other fungal pathogens as well. The majority of pathogenic fungi require iron as an essential growth factor, and elevated free iron levels have been shown to dampen the immune response through impairment phagocytosis [66,67].

Given the potential role of free iron in severe COVID-19, it has recently been theorized that iron chelation could be an effective therapy for hospitalized COVID-19 patients, as iron chelators are safe and effective in reducing free iron levels and possess direct antiviral properties that decrease viral binding and entry via host cell receptors [65]. If iron chelation were to be explored as a potential therapy, it would be wise to consider the effects of various iron chelators on the risk for COVID-19-associated Mucormycosis infections. While chelators such as deferiprone and deferasirox have been shown to protect mice with DKA from *R. oryzae* infection, others, such as deferoxamine, are used by *R. oryzae* as xinosideriphores, and thus, would further predispose patients to Mucormycosis [69,70,71]. It is thus more reasonable, at least from a fungal infection perspective, to consider deferiprone or deferasirox as potential therapies for COVID-19 rather than deferoxamine.

### 4.5. Mechanical Ventilation

Patients receiving mechanical ventilation are at increased risk for bacterial and fungal infections due to the increased rate of microaspiration of contaminated oropharyngeal secretions and gastric contents [72]. This increased introduction of pathogenic microbes likely works synergistically with the above-described risk factors to increase critically ill COVID-19 patients’ susceptibility to fungal infections.

One study analyzing 197 critically ill COVID-19 patients in the ICU and placed on ventilators found 68% of such patients to have positive respiratory fungal cultures, all of which were due to superinfections as each patient had a previously negative fungal culture. *Candida* species represented the most frequently isolated fungi (75.4%), followed by molds including *Aspergillus* (16.4%) and *Mucor* (8.2%) species [73]. Though many had positive fungal respiratory cultures, it is unclear what percentage of patients, if any, actually developed invasive fungal infections in this study. Another study looking at 145 COVID-19 patients admitted to the ICU and placed on ventilators found that 4.8% of these patients developed invasive pulmonary fungal infections, the vast majority of which were *Aspergillus infections* [74].

Of note, these studies cannot give us a relative risk of intubation or prolonged ventilation for the development of fungal respiratory colonization or invasive fungal infections because no non-ventilated patients were studied as controls. Nevertheless, it is likely that the increased introduction of pathogenic microbes through intubation works synergistically with the previously described risk factors to increase critically ill COVID patients’ susceptibility to fungal infections.

### 4.6. T-Cell Lymphopenia

Patients with COVID-19 have been shown to have significantly reduced CD4^+,^ CD8^+,^ and total T-cell counts. The reduction is particularly marked in severe COVID-19 cases, and a strong negative correlation is seen between T-cell counts and IL-6, IL-10, and TNF-α concentrations. The remaining T-cells in COVID-19 patients are also found to be functionally exhausted [12]. T-cells are known to play a vital role in the adaptive immune response against fungal infections, with both CD4+ and CD8^+^ cells known to be particularly vital in the host defense against *Candida* species. Furthermore, imbalances between the Th1 and Th2 subtypes of CD4+ cells seem to predispose individuals to *Aspergillus* infections [75]. These findings suggest that T-Cell lymphopenia is yet another likely predisposing factor in developing secondary fungal infections in COVID-19 patients.

## 5. Fungal Coinfections

### 5.1. Aspergillosis

Aspergillosis infections typically occur in immunocompromised individuals. Risk factors for invasive aspergillosis include corticosteroid therapy, viral infections, and lymphopenia, amongst others [76]. Invasive pulmonary aspergillosis (IPA) has previously been observed in patients with influenza and has been shown to cause more severe disease and increased mortality when compared to patients who had influenza without invasive pulmonary aspergillosis [77]. There are immunopathological similarities between influenza and COVID-19, such as cytokine storm syndrome, tissue damage, lymphopenia, and impaired coagulation [78]. As the COVID-19 pandemic continues to spread, many patients are at risk of coinfection with aspergillosis, which may be difficult to diagnose and worsen patient outcomes. In one study, clinicians evaluated the incidence of IPA in 108 patients with severe COVID-19 and found that 27.7% of these patients also developed COVID-19 associated pulmonary aspergillosis (CAPA) (Table 2).

They also found that patients diagnosed with probable CAPA had a significantly higher 30-day mortality rate than patients with COVID-19 who did not meet the criteria for Aspergillosis [79].

COVID-19 may result in damage to the respiratory epithelium, allowing aspergillosis to invade tissue [80]. Treatment with steroids may be a risk factor for patients with CAPA as one study found that three out of a total of five patients with CAPA were treated with steroids, and all three of them died, while the other two who did not receive steroid treatment remained alive [81]. An evaluation of COVID-19 intensive care patients found a strong association between the use of high dose systemic corticosteroids and aspergillus coinfection [82]. A study observing patients with COVID-19 who were admitted to an intensive care unit found an association with receiving Azithromycin for 3 or more days and being diagnosed with probable IPA. Researchers proposed that as Azithromycin has immunomodulatory properties, its use may be a risk factor for developing IPA in patients with COVID-19 [83].

Moreover, IL-6 is a proinflammatory cytokine with significantly elevated levels in severe COVID-19 patients and has also been found to play a role in protection against *Aspergillus* [84]. Tocilizumab is approved for use in patients with COVID-19 as it is a potent IL-6 inhibitor. While Tocilizumab may be used to treat COVID-19, it may promote a secondary coinfection, such as aspergillosis, as it functions to reduce serum IL-6 levels [85]. While these therapies have been shown to contribute to coinfections, there have been investigations into other therapies. A 2021 paper evaluated the use of thymosin alpha 1 (Tα1), all-trans-retinoic acid (ATRA), and lactoferrin against opportunistic fungal infection [86]. Tα1 demonstrated a protective effect against *Aspergillus fumigatus* in an experimental murine model of bone marrow transplantation. Tα1 increased the Th1 immune response against *Aspergillus fumigatus* [86]. ATRA inhibits in vitro growth of *Aspergillus fumigatus* via enhancing macrophage phagocytosis. ATRA also showed a synergistic effect with antifungal drugs such as amphotericin B and Posaconazole [86]. Lactoferrin demonstrated antifungal activity against *Aspergillus fumigatus* via a possible mechanism of iron sequestration and inducing direct cell membrane damage [86]. The use of steroids, Azithromycin, and Tocilizumab in patients with severe COVID-19 should be monitored closely as such therapies can lead to an *Aspergillus* coinfection. More research is needed to investigate the clinical use of natural immunomodulators further.

### 5.2. Candidiasis

Candidiasis is a fungal infection caused by the yeast *Candida*. *Candida* commonly lives on the skin and mucosal surfaces, including the oropharynx, intestinal lining, and urinary tract. Candida are typically commensal fungal species; however, if certain conditions are met, they can become invasive and cause candidiasis [87]. Hospitals across several countries have observed COVID-19 associated candidiasis (CAC). One study found that Candida sp. was the most prevalent COVID-19 associated fungal coinfection making up 18.8% of such cases [19]. A review found that the prevalence of CAC ranged from 0.7% to 23.5% across several countries [88]. A CAC outbreak in ICUs at a hospital in Mexico resulted in a mortality of 83.3% among patients with candidemia [89]. Understanding the factors associated with driving CAC can protect COVID-19 patients from candida coinfections, in addition to allowing clinicians to promptly diagnose and treat CAC.

Researchers from Iran found that COVID-19 patients with oropharyngeal candidiasis (OPC) had at least one of the following risk factors: lymphocytopenia, ICU admission, mechanical ventilation, corticosteroid use, broad-spectrum antibiotic use, or an immunocompromised condition (Table 2). Of these risk factors, broad-spectrum antibiotic use was the most common as it was present in 92.5% of OPC patients [90]. As antibiotic use may disrupt the balance between oral bacterial and yeast populations, it can create an environment that permits *candida* overgrowth and infection [91]. Clinicians in Turkey observed that COVID-19 patients who were receiving broad-spectrum antibiotics were at an increased risk for developing candidemia [92]. In COVID-19 treatment guidelines, the World Health Organization recommends against the use of broad-spectrum antibiotics unless there is a clinical suspicion of bacterial infection [93]. A study done in Italy found that three critically ill patients with COVID-19 developed candidemia after treatment with Tocilizumab, an IL-6 inhibitor [94]. A trial assessing the susceptibility of candidiasis in IL-6 deficient mice found that IL-6 deficient mice had increased mortality and higher *candida* fungal loads when compared to control mice [95].

A case-level analysis found that 25.5% of patients with candidemia were also positive for COVID-19 and that ICU admission, mechanical ventilation, catheter placement, steroid and immunosuppressant use were 1.3 times more common in these patients when compared to patients who had candidemia but were COVID-19 negative [96]. Arastehfar et al. also found that 74.5% of patients with COVID-19 associated candidemia infections had undergone central venous catheterization [88]. Catheter placement has been implicated in the introduction and proliferation of microorganisms such as *Candida*, which pose therapeutic problems as they can form biofilms [97]. *Candida* colonization is common in mechanically ventilated patients, as long-term ventilation is associated with a significant increase in respiratory and urinary tract *candida* populations [98,99]. As previously mentioned, Gaziano et al. demonstrated the potential of various natural immunomodulators (Tα1, ATRA, and Lactoferrin) against opportunistic fungal coinfections [86]. In vivo and in vitro experimental studies showed the remarkable antifungal activity of Tα1 against systemic *Candida albicans* infection. Tα1 potentiates polymorphonuclear cell-induced intracellular killing of the fungus [86]. In vitro, ATRA can be used as a fungistatic drug by inhibiting the growth of *Candida albicans*. Lactoferrin, through its ability to sequester iron, showed strong antifungal activity [86]. Clinicians must be aware of risk factors for nosocomial candidiasis coinfections in patients with COVID-19, particularly when such patients are treated with broad-spectrum antibiotics, corticosteroids, Tocilizumab, especially in a background of catheter placement or mechanical ventilation. More research is needed to further investigate the clinical use of natural immunomodulators.

### 5.3. Cryptococcosis

Cryptococcosis is a fungal infection caused by *cryptococcus* species and can be fatal in immunocompromised individuals. It is one of the more prevalent infections in patients with HIV and AIDS [100]. Previous data found that 81% of patients with cryptococcosis developed sepsis and that the 30-day fatality rate of such cases was 37% [101]. To date, there have only been a handful of case reports on COVID-19 associated cryptococcosis infections (Table 2). While it appears to be a rare occurrence, investigating potential risk factors and causes for COVID-19 associated cryptococcosis is important. The infection can quickly become fatal if not identified and treated appropriately, especially in immunocompromised individuals with HIV/AIDS.

In one case report, a patient with a history of kidney transplant and liver cirrhosis who was COVID-19 positive later developed a cryptococcus coinfection and did not survive. The researchers suggest that CD4+ T-cell depletion caused by COVID-19 may have been a key driver for cryptococcosis in this case; however, they could not draw a definitive conclusion as cryptococcal infections have also been associated with both solid organ transplant and liver cirrhosis patients independent of COVID-19 [102]. Another case report found that a COVID-19 positive patient treated with Tocilizumab and corticosteroids went on to develop cryptococcemia and died within 10 days [103]. Previous research has shown an association between high levels of IL-6 and resistance to cryptococcal infection [104]. The most recent case report presented a patient with COVID-19 who was treated with dexamethasone and developed severe cryptococcal meningitis [105]. The authors suggest that the impact of steroids on T-cell function should be further investigated, as T-cell depletion has been shown to be a driving factor for cryptococcal meningitis.

### 5.4. Mucormycosis

Mucormycosis is an infection caused by fungi in the order of Mucorales, most frequently by the *Rhizopus* spp., *Lichtheimia* spp. and *Mucor* spp., which account for nearly 75% of all cases [106]. The most common route of infection is through inhalation of spores that lead to pulmonary infection, typically in immunocompromised individuals. Cutaneous and soft-tissue manifestations may also be common, and in diabetic populations, rhino-orbital mucormycosis is commonly presented. In a meta-analysis, diabetes mellitus was the most common comorbidity contributing to the development of rhino-orbital mucormycosis in 340 of 851 (40%) patients with an odds ratio of 2.49 (95% CI 1.77–3.54) compared to the next possible factor of having hematological malignancies with an odds ratio of 0.76 (0.44–1.26) [106,107]. Risk factors for mucormycosis include diabetic ketoacidosis, corticosteroid treatment, organ/bone marrow transplantation, neutropenia, trauma/burns, and elevated levels of free iron [30,108].

Mucormycosis, popularly known as black fungal infection, is an emerging disease, with the occurrence in the general population cited as 0.005 to 1.7 per million [104]. However, in India, the prevalence of mucormycosis is close to 0.14 cases per 1000 population, nearly 80 times its prevalence in developed countries [109]. The surge COVID-19 cases in India had been associated with increased reports of invasive mucormycosis post-COVID-19 and are continuously being reported to be rising [110]. While many treatment options have been evaluated for COVID-19, glucocorticoids have been shown to improve survival but, on the other hand, can lead to secondary fungal infections (Table 2). The combination of SARS-CoV-2, steroid overuse, and uncontrolled diabetes mellitus has contributed to a significant increase in the incidence of invasive mucormycosis [68]. Another contributing virulence factor that plays an important role in the pathogenesis of mucormycosis is its ability to uptake free unbound iron from the host. Hyper-ferritinemic states such as diabetic ketoacidosis, iron-chelator treatment in dialysis, or severe COVID-19 can further predispose an individual to mucormycosis [54,63,64,111].

Treatment for mucormycosis involves surgical debridement whenever possible, in addition to systemic antifungal therapy with liposomal Amphotericin B as the choice of drug [107]. However, despite surgery and antifungal treatment, the overall mortality rate for mucormycosis remains over 50% and approaches 100% in patients with disseminated disease and neutropenia [112]. Thus, COVID-19 treated patients who are diabetic and were administered corticosteroids for controlling the severity of infection may be more susceptible to mucormycosis infections with poor prognosis, further complicating the pandemic scenario by leading to more fatalities.

## 6. Conclusions

COVID-19 is associated with a high number of secondary infections of both fungal and bacterial origin. The combination of diabetes and increased use of corticosteroids to combat infection with COVID-19 appears to increase the risk of development and aggravates existing opportunistic fungal infections. Mechanical ventilation, catheter placement, and immunosuppressant therapies appear to play a role in manifesting various fungal coinfections in COVID-19 patients. Thus, physicians and healthcare professionals should be aware of risk factors and the possibility of secondary infections associated with them in treating COVID-19 patients. Careful measures should be taken in minimizing the dosage and duration for therapeutic agents such as corticosteroids, immunosuppressants, and broad-spectrum antibiotics. Findings from this review identify possible explanations for the increase in fungal coinfections seen in COVID-19 infected patients and help determine the connection between various manifestations of fungal infections, causative agents, and risk factors.

## Figures and Tables

**Table 1:** Comparison of fungal infection and COVID-19 infection via analysis of overlapping and differing symptom presentations. [24,25,26,27,28].

Fungus	Infection	CDC-Main Fungal Symptoms Overlapping with COVID-19	CDC-Main Fungal Symptoms Differing from COVID-19
*Aspergillus genera*	Aspergillosis	Shortness of breath (SOB), cough, fever, fatigue, runny nose, headache (HA), chest pain, congestion, loss of smell	Wheezing, hemoptysis
*Candida auris*	Candidiasis	Fever, chills, loss of taste, sore throat	Odynophagia, oral thrush, vaginal candidiasis
*Cryptococcus neoformans*	Cryptococcosis	Cough, SOB, fever, HA, nausea, vomiting, confusion, chest pain	Light sensitivity
Mucorales order	Mucormycosis	HA, nasal congestion, fever, cough, chest pain, SOB, nausea, vomiting	Unilateral facial swelling, black lesions on nasal bridge or inside the mouth, gastrointestinal (GI) bleeding

**Table 2:** Summary table of fungal coinfection findings by country (n= total number of patients). [19,79,80,81,82,83,85,88,89,92,94,96,102,103,105,106,110].

Author	Country	Type of Fungal Infection	Severity (ICU, Floor, or Mixed)	Study Type	Total Patients (n)	Fungal Co-Infection (%)	Death (%)
Bartoletti et al.	Italy	Aspergillosis	ICU	Prospective	108	27.7	44
Koehler et al.	Germany	Aspergillosis	ICU	Retrospective	19	26.3	60
White et al.	United Kingdom	Aspergillosis	ICU	Prospective	135	14.1	57.9
Dellière et al.	France	Aspergillosis	ICU	Retrospective	366	5.7	71.4
Lai & Yu	Multiple France Germany Netherlands Belgium Italy Austria	Aspergillosis	Mixed	Review	Total: 34 11 7 7 7 1 1	100	64.7
Musuuza et al.	Multiple	Candidiasis	Mixed	Systematic Review and Meta-analysis	N/A	18.8	N/A
Arastehfar et al.	Multiple Spain India Iran Italy United Kingdom China	Candidiasis	Mixed	Review	989 596 1059 43 135 17	0.3 2.5 5 8 12.6 23.5	66.7 60 N/A N/A 47.1 N/A
Villanueva-Loza no et al.	Mexico	Candidiasis	ICU	Retrospective	12	50	83.3
Coşkun et al.	Turkey	Candidiasis	ICU	Retrospective	627	2.6	80
Antinori et al.	Italy	Candidiasis	Mixed	Prospective	43	6.9	N/A
Seagle et al.	United States	Candidiasis	Mixed	Case-level analysis	64	100	60
Passarelli et al.	United States	Cryptococcosis	ICU	Case report	1	100	100
Khatib et al.	Qatar	Cryptococcosis	ICU	Case report	1	100	100
Ghanem & Sivasubramanian	United States	Cryptococcosis	Mixed	Case Report	1	100	0
Pal et al.	Multiple India United States Egypt Iran Brazil Chile United Kingdom France Italy Austria Mexico	Mucormycosis	Mixed	Systematic review and meta-analysis	Total: 99 71 10 6 3 2 2 1 1 1 1 1	100	34
Jeong et al.	Multiple South America Europe Asia Africa Australia/New Zealand	Mucormycosis	Mixed	Systematic review and Meta-analysis	125 172 111 18 21 Total: 447	14	41

## Abbreviations

Severe Acute Respiratory Syndrome Coronavirus 2	(SARS-CoV-2)
Centers for Disease Control	(CDC)
United States	(US)
Interleukin-6	(IL-6)
Nuclear Factor Kappa Beta	(NF-κB)
COVID-19 Associated Mucormycosis	(CAM)
Diabetic Ketoacidosis	(DKA)
Intensive Care Unit	(ICU)
Hypoxia inducible factor-1α	(HIF-1α)
Angiotensin-converting enzyme-2	(ACE-2)
Diabetes Mellitus	(DM)
Tumor necrosis factor-alpha	(TNF-α)
Interleukin-10	(IL-10)
Interferon gamma	(IFN-ℽ)
T helper 1 cell	(Th1)
Invasive Pulmonary Aspergillosis	(IPA)
COVID-19 Associated Pulmonary Aspergillosis	(CAPA)
COVID-19 Associated Candidiasis	(CAC)
Oropharyngeal Candidiasis	(OPC)
Human Immunodeficiency Virus/Acquired Immunodeficiency Syndrome	(HIV/AIDS)
Shortness of breath	(SOB)
Headache	(HA)
Gastrointestinal	(GI)
Thymosin alpha 1	(Tα1)
All Trans Retinoic Acid	(ATRA)
